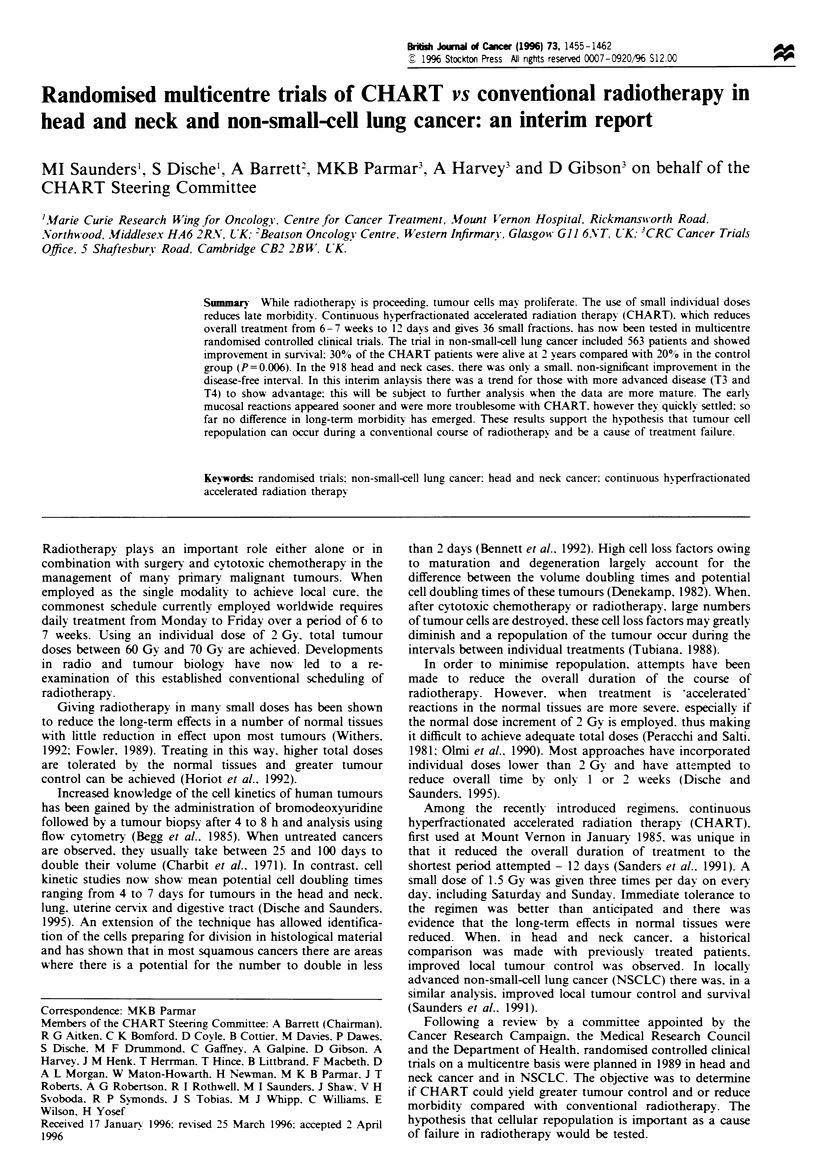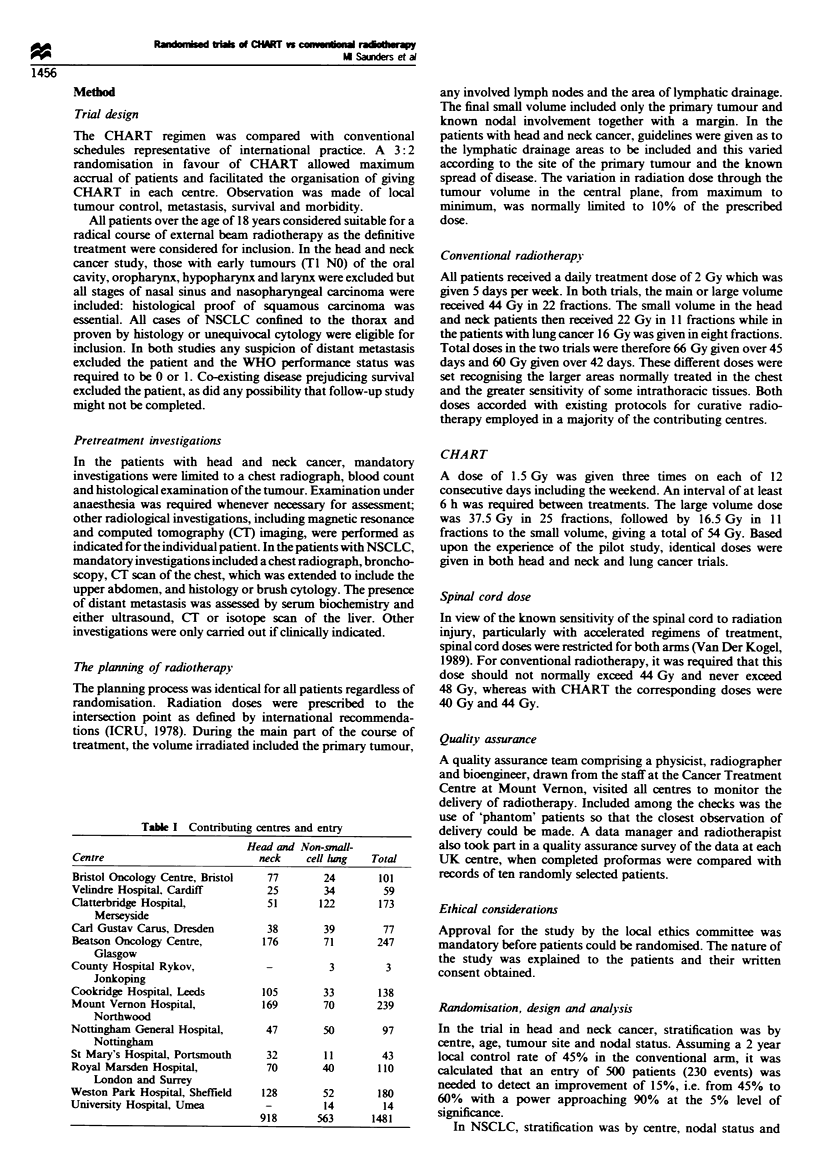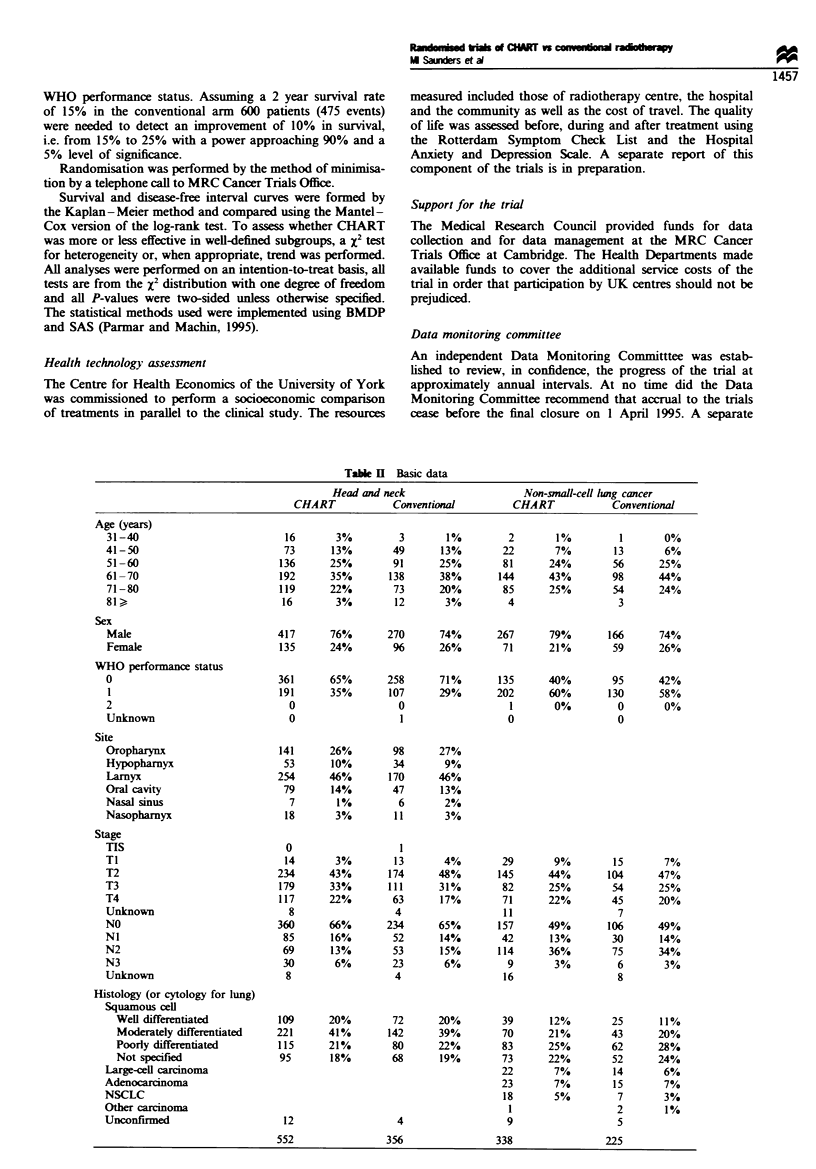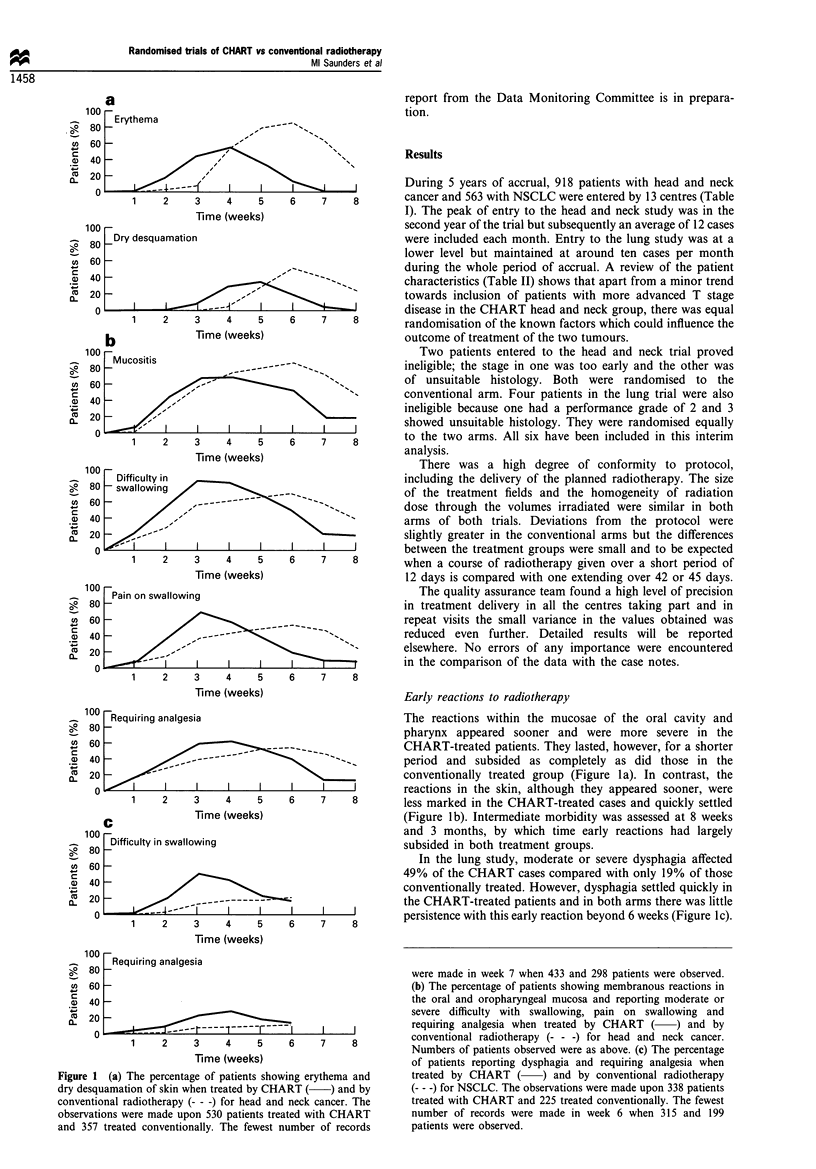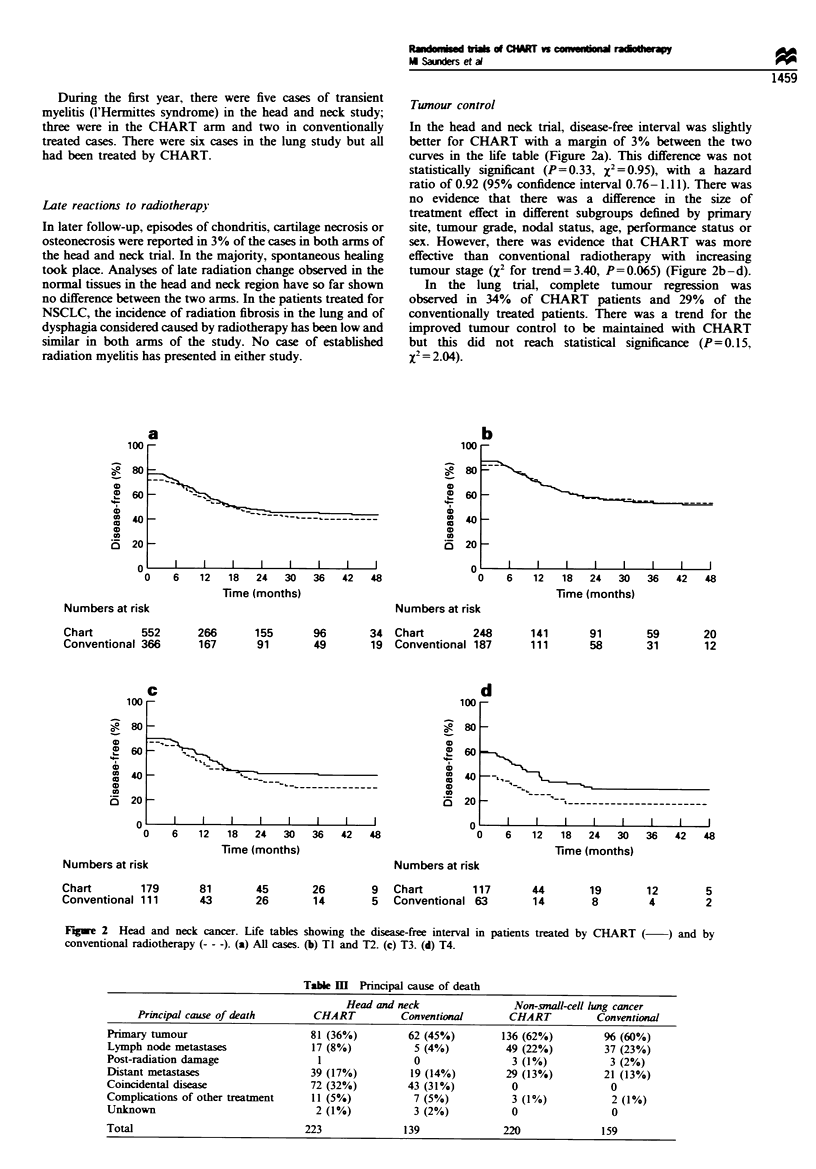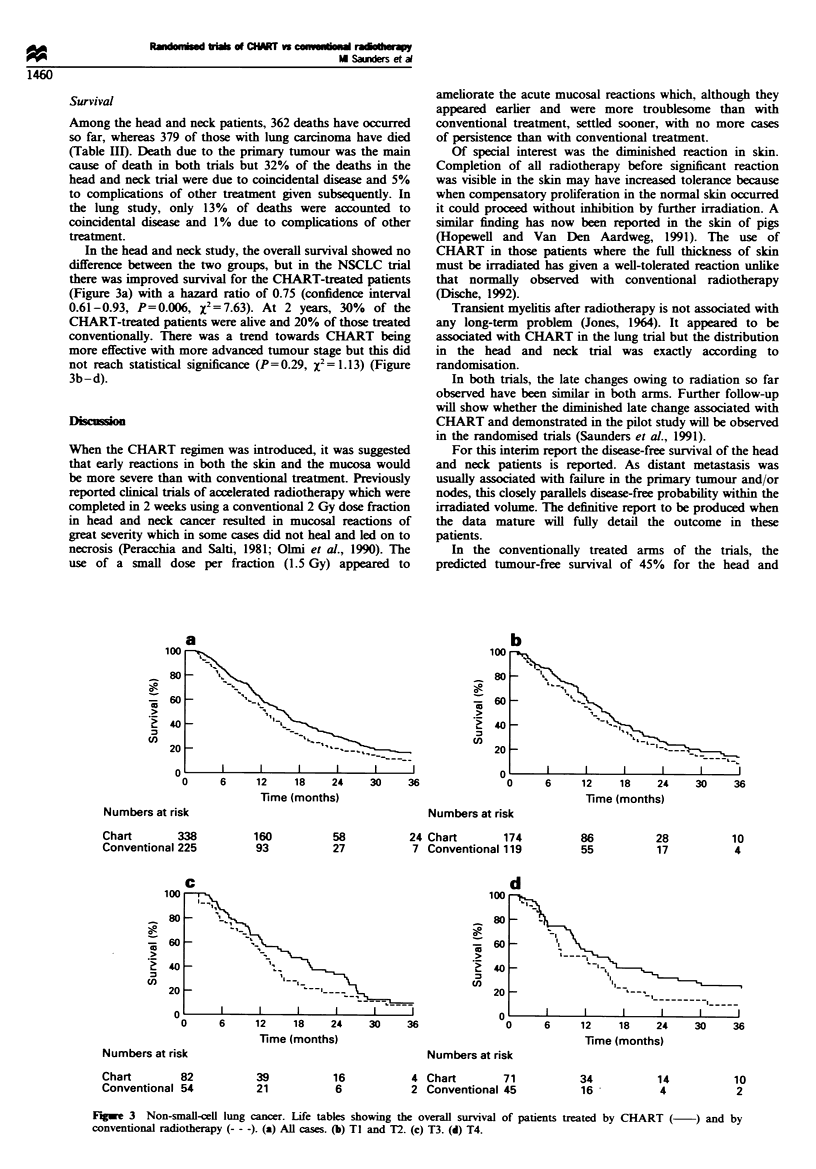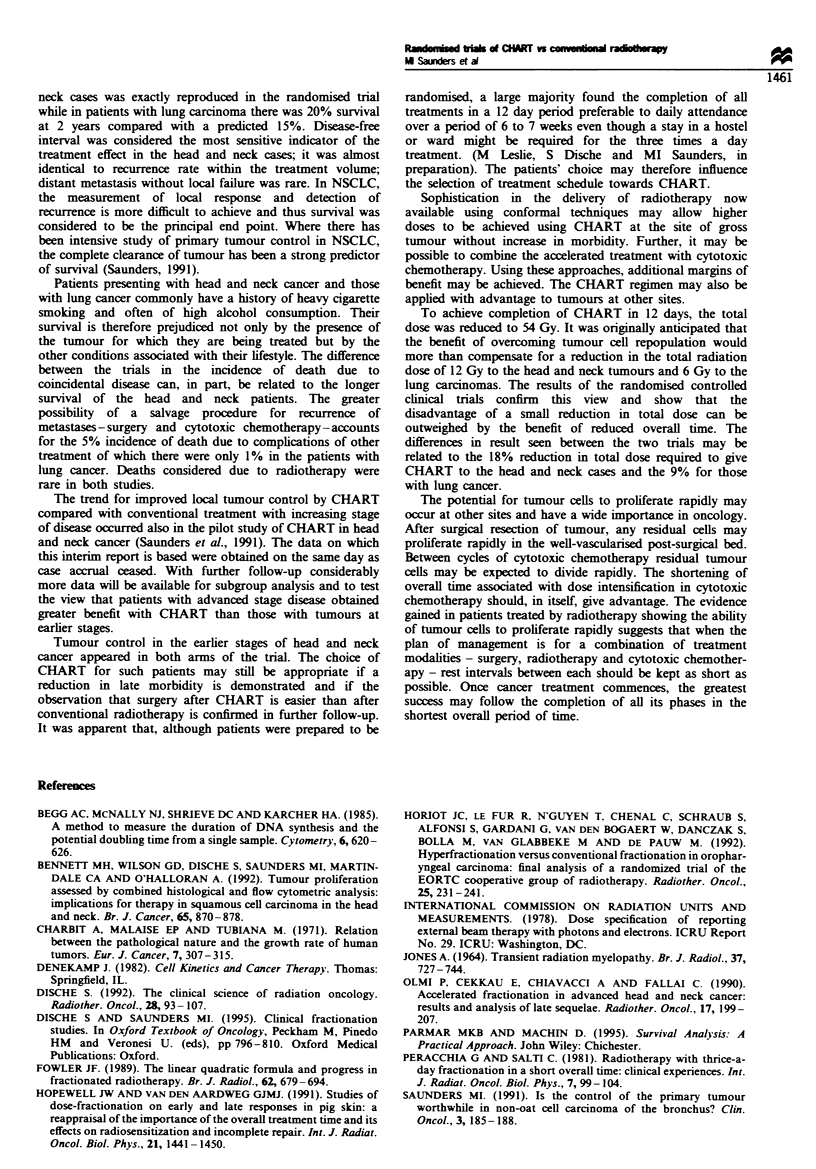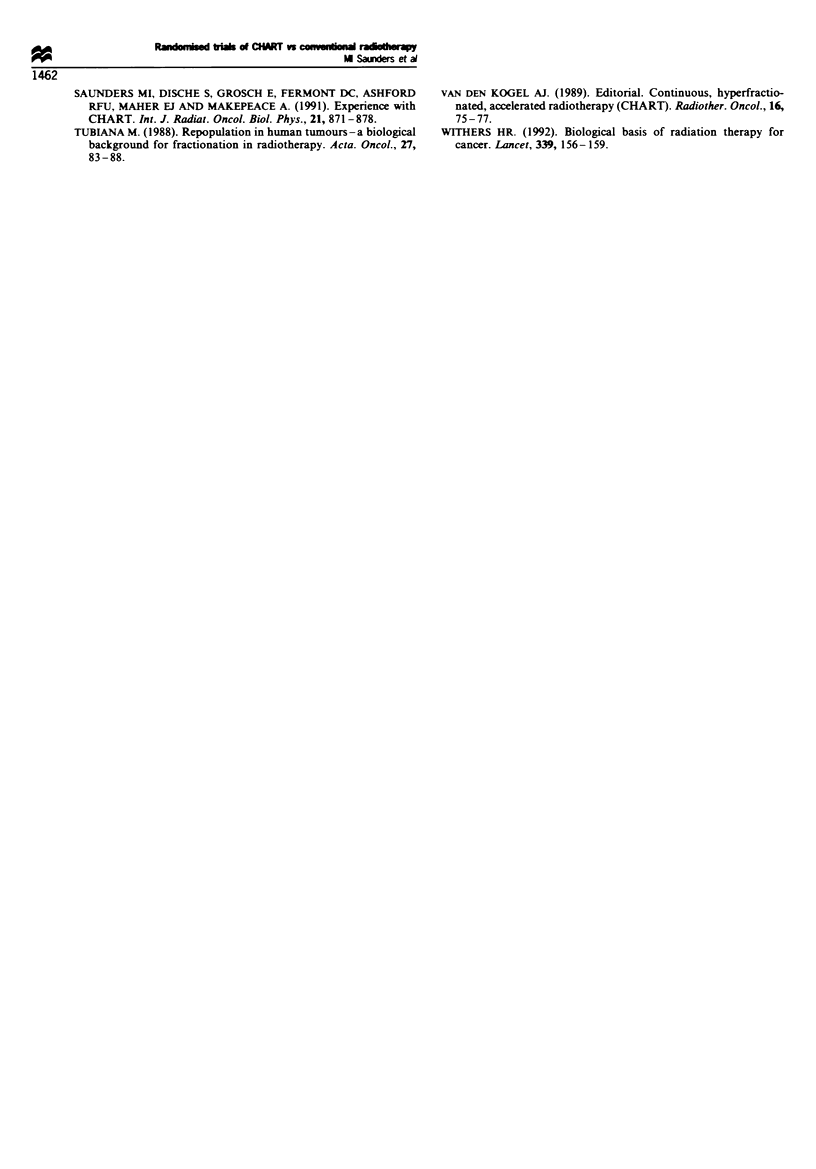# Randomised multicentre trials of CHART vs conventional radiotherapy in head and neck and non-small-cell lung cancer: an interim report. CHART Steering Committee.

**DOI:** 10.1038/bjc.1996.276

**Published:** 1996-06

**Authors:** M. I. Saunders, S. Dische, A. Barrett, M. K. Parmar, A. Harvey, D. Gibson

**Affiliations:** Marie Curie Research Wing for Oncology, Centre for Cancer Treatment, Mount Vernon Hospital, Northwood, Middlesex, UK.

## Abstract

While radiotherapy is proceeding, tumour cells may proliferate. The use of small individual doses reduces late morbidity. Continuous hyperfractionated accelerated radiation therapy (CHART), which reduces overall treatment from 6-7 weeks to 12 days and gives 36 small fractions, has now been tested in multicentre randomised controlled clinical trials. The trial in non-small-cell lung cancer included 563 patients and showed improvement in survival; 30% of the CHART patients were alive at 2 years compared with 20% in the control group (P = 0.006). In the 918 head and neck cases, there was only a small, non-significant improvement in the disease-free interval. In this interim analysis there was a trend for those with more advanced disease (T3 and T4) to show advantage; this will be subject to further analysis when the data are more mature. The early mucosal reactions appeared sooner and were more troublesome with CHART, however they quickly settled; so far no difference in long-term morbidity has emerged. These results support the hypothesis that tumour cell repopulation can occur during a conventional course of radiotherapy and be a cause of treatment failure.


British Journal of Cancer (1996) 73, 1455-1462

© 1996 Stockton Press All rights reserved 0007-0920/96 $12.00

### Randomised multicentre trials of CHART *vs* conventional radiotherapy in head and neck and non-small-cell lung cancer: an interim report

MI Saunders[1], S Dische[1], A Barrett[2], MKB Parmar[3], A Harvey[3] and D Gibson[3] on behalf of the CHART Steering Committee

[1]*Marie Curie Research Wing for Oncology, Centre for Cancer Treatment, Mount Vernon Hospital, Rickmansworth Road, Northwood, Middlesex HA6 2RN, UK;* [2]*Beatson Oncology Centre, Western Infirmary, Glasgow G11 6NT, UK;* [3]*CRC Cancer Trials Office, 5 Shaftesbury Road, Cambridge CB2 2BW, UK.*

**Summary** While radiotherapy is proceeding, tumour cells may proliferate. The use of small individual doses reduces late morbidity. Continuous hyperfractionated accelerated radiation therapy (CHART), which reduces overall treatment from 6–7 weeks to 12 days and gives 36 small fractions, has now been tested in multicentre randomised controlled clinical trials. The trial in non-small-cell lung cancer included 563 patients and showed improvement in survival: 30% of the CHART patients were alive at 2 years compared with 20% in the control group ($P = 0.006$). In the 918 head and neck cases, there was only a small, non-significant improvement in the disease-free interval. In this interim anlaysis there was a trend for those with more advanced disease (T3 and T4) to show advantage: this will be subject to further analysis when the data are more mature. The early mucosal reactions appeared sooner and were more troublesome with CHART, however they quickly settled: so far no difference in long-term morbidity has emerged. These results support the hypothesis that tumour cell repopulation can occur during a conventional course of radiotherapy and be a cause of treatment failure.

**Keywords:** randomised trials; non-small-cell lung cancer; head and neck cancer; continuous hyperfractionated accelerated radiation therapy

Radiotherapy plays an important role either alone or in combination with surgery and cytotoxic chemotherapy in the management of many primary malignant tumours. When employed as the single modality to achieve local cure, the commonest schedule currently employed worldwide requires daily treatment from Monday to Friday over a period of 6 to 7 weeks. Using an individual dose of 2 Gy, total tumour doses between 60 Gy and 70 Gy are achieved. Developments in radio and tumour biology have now led to a re-examination of this established conventional scheduling of radiotherapy.

Giving radiotherapy in many small doses has been shown to reduce the long-term effects in a number of normal tissues with little reduction in effect upon most tumours (Withers, 1992; Fowler, 1989). Treating in this way, higher total doses are tolerated by the normal tissues and greater tumour control can be achieved (Horiot *et al.*, 1992).

Increased knowledge of the cell kinetics of human tumours has been gained by the administration of bromodeoxyuridine followed by a tumour biopsy after 4 to 8 h and analysis using flow cytometry (Begg *et al.*, 1985). When untreated cancers are observed, they usually take between 25 and 100 days to double their volume (Charbit *et al.*, 1971). In contrast, cell kinetic studies now show mean potential cell doubling times ranging from 4 to 7 days for tumours in the head and neck, lung, uterine cervix and digestive tract (Dische and Saunders, 1995). An extension of the technique has allowed identification of the cells preparing for division in histological material and has shown that in most squamous cancers there are areas where there is a potential for the number to double in less than 2 days (Bennett *et al.*, 1992). High cell loss factors owing to maturation and degeneration largely account for the difference between the volume doubling times and potential cell doubling times of these tumours (Denekamp, 1982). When, after cytotoxic chemotherapy or radiotherapy, large numbers of tumour cells are destroyed, these cell loss factors may greatly diminish and a repopulation of the tumour occur during the intervals between individual treatments (Tubiana, 1988).

In order to minimise repopulation, attempts have been made to reduce the overall duration of the course of radiotherapy. However, when treatment is 'accelerated' reactions in the normal tissues are more severe, especially if the normal dose increment of 2 Gy is employed, thus making it difficult to achieve adequate total doses (Peracchi and Salti, 1981; Olmi *et al.*, 1990). Most approaches have incorporated individual doses lower than 2 Gy and have attempted to reduce overall time by only 1 or 2 weeks (Dische and Saunders, 1995).

Among the recently introduced regimens, continuous hyperfractionated accelerated radiation therapy (CHART), first used at Mount Vernon in January 1985, was unique in that it reduced the overall duration of treatment to the shortest period attempted – 12 days (Sanders *et al.*, 1991). A small dose of 1.5 Gy was given three times per day on every day, including Saturday and Sunday. Immediate tolerance to the regimen was better than anticipated and there was evidence that the long-term effects in normal tissues were reduced. When, in head and neck cancer, a historical comparison was made with previously treated patients, improved local tumour control was observed. In locally advanced non-small-cell lung cancer (NSCLC) there was, in a similar analysis, improved local tumour control and survival (Saunders *et al.*, 1991).

Following a review by a committee appointed by the Cancer Research Campaign, the Medical Research Council and the Department of Health, randomised controlled clinical trials on a multicentre basis were planned in 1989 in head and neck cancer and in NSCLC. The objective was to determine if CHART could yield greater tumour control and/or reduce morbidity compared with conventional radiotherapy. The hypothesis that cellular repopulation is important as a cause of failure in radiotherapy would be tested.

Correspondence: MKB Parmar
Members of the CHART Steering Committee: A Barrett (Chairman), R G Aitken, C K Bomford, D Coyle, B Cottier, M Davies, P Dawes, S Dische, M F Drummond, C Gaffney, A Galpine, D Gibson, A Harvey, J M Henk, T Herrman, T Hince, B Littbrand, F Macbeth, D A L Morgan, W Maton-Howarth, H Newman, M K B Parmar, J T Roberts, A G Robertson, R I Rothwell, M I Saunders, J Shaw, V H Svoboda, R P Symonds, J S Tobias, M J Whipp, C Williams, E Wilson, H Yosef
Received 17 January 1996; revised 25 March 1996; accepted 2 April 1996

### Method

### Trial design

The CHART regimen was compared with conventional schedules representative of international practice. A 3:2 randomisation in favour of CHART allowed maximum accrual of patients and facilitated the organisation of giving CHART in each centre. Observation was made of local tumour control, metastasis, survival and morbidity.

All patients over the age of 18 years considered suitable for a radical course of external beam radiotherapy as the definitive treatment were considered for inclusion. In the head and neck cancer study, those with early tumours (T1 N0) of the oral cavity, oropharynx, hypopharynx and larynx were excluded but all stages of nasal sinus and nasopharyngeal carcinoma were included: histological proof of squamous carcinoma was essential. All cases of NSCLC confined to the thorax and proven by histology or unequivocal cytology were eligible for inclusion. In both studies any suspicion of distant metastasis excluded the patient and the WHO performance status was required to be 0 or 1. Co-existing disease prejudicing survival excluded the patient, as did any possibility that follow-up study might not be completed.

### Pretreatment investigations

In the patients with head and neck cancer, mandatory investigations were limited to a chest radiograph, blood count and histological examination of the tumour. Examination under anaesthesia was required whenever necessary for assessment; other radiological investigations, including magnetic resonance and computed tomography (CT) imaging, were performed as indicated for the individual patient. In the patients with NSCLC, mandatory investigations included a chest radiograph, bronchoscopy, CT scan of the chest, which was extended to include the upper abdomen, and histology or brush cytology. The presence of distant metastasis was assessed by serum biochemistry and either ultrasound, CT or isotope scan of the liver. Other investigations were only carried out if clinically indicated.

### The planning of radiotherapy

The planning process was identical for all patients regardless of randomisation. Radiation doses were prescribed to the intersection point as defined by international recommendations (ICRU, 1978). During the main part of the course of treatment, the volume irradiated included the primary tumour, any involved lymph nodes and the area of lymphatic drainage. The final small volume included only the primary tumour and known nodal involvement together with a margin. In the patients with head and neck cancer, guidelines were given as to the lymphatic drainage areas to be included and this varied according to the site of the primary tumour and the known spread of disease. The variation in radiation dose through the tumour volume in the central plane, from maximum to minimum, was normally limited to 10% of the prescribed dose.

**Table I** Contributing centres and entry

| Centre | Head and neck | Non-small-cell lung | Total |
|---|---|---|---|
| Bristol Oncology Centre, Bristol | 77 | 24 | 101 |
| Velindre Hospital, Cardiff | 25 | 34 | 59 |
| Clatterbridge Hospital, Merseyside | 51 | 122 | 173 |
| Carl Gustav Carus, Dresden | 38 | 39 | 77 |
| Beatson Oncology Centre, Glasgow | 176 | 71 | 247 |
| County Hospital Rykov, Jonkoping | – | 3 | 3 |
| Cookridge Hospital, Leeds | 105 | 33 | 138 |
| Mount Vernon Hospital, Northwood | 169 | 70 | 239 |
| Nottingham General Hospital, Nottingham | 47 | 50 | 97 |
| St Mary's Hospital, Portsmouth | 32 | 11 | 43 |
| Royal Marsden Hospital, London and Surrey | 70 | 40 | 110 |
| Weston Park Hospital, Sheffield | 128 | 52 | 180 |
| University Hospital, Umea | – | 14 | 14 |
| | 918 | 563 | 1481 |

### Conventional radiotherapy

All patients received a daily treatment dose of 2 Gy which was given 5 days per week. In both trials, the main or large volume received 44 Gy in 22 fractions. The small volume in the head and neck patients then received 22 Gy in 11 fractions while in the patients with lung cancer 16 Gy was given in eight fractions. Total doses in the two trials were therefore 66 Gy given over 45 days and 60 Gy given over 42 days. These different doses were set recognising the larger areas normally treated in the chest and the greater sensitivity of some intrathoracic tissues. Both doses accorded with existing protocols for curative radiotherapy employed in a majority of the contributing centres.

### CHART

A dose of 1.5 Gy was given three times on each of 12 consecutive days including the weekend. An interval of at least 6 h was required between treatments. The large volume dose was 37.5 Gy in 25 fractions, followed by 16.5 Gy in 11 fractions to the small volume, giving a total of 54 Gy. Based upon the experience of the pilot study, identical doses were given in both head and neck and lung cancer trials.

### Spinal cord dose

In view of the known sensitivity of the spinal cord to radiation injury, particularly with accelerated regimens of treatment, spinal cord doses were restricted for both arms (Van Der Kogel, 1989). For conventional radiotherapy, it was required that this dose should not normally exceed 44 Gy and never exceed 48 Gy, whereas with CHART the corresponding doses were 40 Gy and 44 Gy.

### Quality assurance

A quality assurance team comprising a physicist, radiographer and bioengineer, drawn from the staff at the Cancer Treatment Centre at Mount Vernon, visited all centres to monitor the delivery of radiotherapy. Included among the checks was the use of 'phantom' patients so that the closest observation of delivery could be made. A data manager and radiotherapist also took part in a quality assurance survey of the data at each UK centre, when completed proformas were compared with records of ten randomly selected patients.

### Ethical considerations

Approval for the study by the local ethics committee was mandatory before patients could be randomised. The nature of the study was explained to the patients and their written consent obtained.

### Randomisation, design and analysis

In the trial in head and neck cancer, stratification was by centre, age, tumour site and nodal status. Assuming a 2 year local control rate of 45% in the conventional arm, it was calculated that an entry of 500 patients (230 events) was needed to detect an improvement of 15%, i.e. from 45% to 60% with a power approaching 90% at the 5% level of significance.

In NSCLC, stratification was by centre, nodal status and

WHO performance status. Assuming a 2 year survival rate of 15% in the conventional arm 600 patients (475 events) were needed to detect an improvement of 10% in survival, i.e. from 15% to 25% with a power approaching 90% and a 5% level of significance.

Randomisation was performed by the method of minimisation by a telephone call to MRC Cancer Trials Office.

Survival and disease-free interval curves were formed by the Kaplan–Meier method and compared using the Mantel–Cox version of the log-rank test. To assess whether CHART was more or less effective in well-defined subgroups, a $\chi^2$ test for heterogeneity or, when appropriate, trend was performed. All analyses were performed on an intention-to-treat basis, all tests are from the $\chi^2$ distribution with one degree of freedom and all $P$-values were two-sided unless otherwise specified. The statistical methods used were implemented using BMDP and SAS (Parmar and Machin, 1995).

### *Health technology assessment*

The Centre for Health Economics of the University of York was commissioned to perform a socioeconomic comparison of treatments in parallel to the clinical study. The resources measured included those of radiotherapy centre, the hospital and the community as well as the cost of travel. The quality of life was assessed before, during and after treatment using the Rotterdam Symptom Check List and the Hospital Anxiety and Depression Scale. A separate report of this component of the trials is in preparation.

### *Support for the trial*

The Medical Research Council provided funds for data collection and for data management at the MRC Cancer Trials Office at Cambridge. The Health Departments made available funds to cover the additional service costs of the trial in order that participation by UK centres should not be prejudiced.

### *Data monitoring committee*

An independent Data Monitoring Committtee was established to review, in confidence, the progress of the trial at approximately annual intervals. At no time did the Data Monitoring Committee recommend that accrual to the trials cease before the final closure on 1 April 1995. A separate

**Table II** Basic data

| | *Head and neck* | | | | *Non-small-cell lung cancer* | | | |
|---|---|---|---|---|---|---|---|---|
| | *CHART* | | *Conventional* | | *CHART* | | *Conventional* | |
| Age (years) | | | | | | | | |
| 31–40 | 16 | 3% | 3 | 1% | 2 | 1% | 1 | 0% |
| 41–50 | 73 | 13% | 49 | 13% | 22 | 7% | 13 | 6% |
| 51–60 | 136 | 25% | 91 | 25% | 81 | 24% | 56 | 25% |
| 61–70 | 192 | 35% | 138 | 38% | 144 | 43% | 98 | 44% |
| 71–80 | 119 | 22% | 73 | 20% | 85 | 25% | 54 | 24% |
| 81⩾ | 16 | 3% | 12 | 3% | 4 | | 3 | |
| Sex | | | | | | | | |
| Male | 417 | 76% | 270 | 74% | 267 | 79% | 166 | 74% |
| Female | 135 | 24% | 96 | 26% | 71 | 21% | 59 | 26% |
| WHO performance status | | | | | | | | |
| 0 | 361 | 65% | 258 | 71% | 135 | 40% | 95 | 42% |
| 1 | 191 | 35% | 107 | 29% | 202 | 60% | 130 | 58% |
| 2 | 0 | | 0 | | 1 | 0% | 0 | 0% |
| Unknown | 0 | | 1 | | 0 | | 0 | |
| Site | | | | | | | | |
| Oropharynx | 141 | 26% | 98 | 27% | | | | |
| Hypopharnyx | 53 | 10% | 34 | 9% | | | | |
| Larnyx | 254 | 46% | 170 | 46% | | | | |
| Oral cavity | 79 | 14% | 47 | 13% | | | | |
| Nasal sinus | 7 | 1% | 6 | 2% | | | | |
| Nasopharnyx | 18 | 3% | 11 | 3% | | | | |
| Stage | | | | | | | | |
| TIS | 0 | | 1 | | | | | |
| T1 | 14 | 3% | 13 | 4% | 29 | 9% | 15 | 7% |
| T2 | 234 | 43% | 174 | 48% | 145 | 44% | 104 | 47% |
| T3 | 179 | 33% | 111 | 31% | 82 | 25% | 54 | 25% |
| T4 | 117 | 22% | 63 | 17% | 71 | 22% | 45 | 20% |
| Unknown | 8 | | 4 | | 11 | | 7 | |
| N0 | 360 | 66% | 234 | 65% | 157 | 49% | 106 | 49% |
| N1 | 85 | 16% | 52 | 14% | 42 | 13% | 30 | 14% |
| N2 | 69 | 13% | 53 | 15% | 114 | 36% | 75 | 34% |
| N3 | 30 | 6% | 23 | 6% | 9 | 3% | 6 | 3% |
| Unknown | 8 | | 4 | | 16 | | 8 | |
| Histology (or cytology for lung) | | | | | | | | |
| Squamous cell | | | | | | | | |
| Well differentiated | 109 | 20% | 72 | 20% | 39 | 12% | 25 | 11% |
| Moderately differentiated | 221 | 41% | 142 | 39% | 70 | 21% | 43 | 20% |
| Poorly differentiated | 115 | 21% | 80 | 22% | 83 | 25% | 62 | 28% |
| Not specified | 95 | 18% | 68 | 19% | 73 | 22% | 52 | 24% |
| Large-cell carcinoma | | | | | 22 | 7% | 14 | 6% |
| Adenocarcinoma | | | | | 23 | 7% | 15 | 7% |
| NSCLC | | | | | 18 | 5% | 7 | 3% |
| Other carcinoma | | | | | 1 | | 2 | 1% |
| Unconfirmed | 12 | | 4 | | 9 | | 5 | |
| | 552 | | 356 | | 338 | | 225 | |

**Figure 1** (**a**) The percentage of patients showing erythema and dry desquamation of skin when treated by CHART (——) and by conventional radiotherapy (- - -) for head and neck cancer. The observations were made upon 530 patients treated with CHART and 357 treated conventionally. The fewest number of records were made in week 7 when 433 and 298 patients were observed. (**b**) The percentage of patients showing membranous reactions in the oral and oropharyngeal mucosa and reporting moderate or severe difficulty with swallowing, pain on swallowing and requiring analgesia when treated by CHART (——) and by conventional radiotherapy (- - -) for head and neck cancer. Numbers of patients observed were as above. (**c**) The percentage of patients reporting dysphagia and requiring analgesia when treated by CHART (——) and by conventional radiotherapy (- - -) for NSCLC. The observations were made upon 338 patients treated with CHART and 225 treated conventionally. The fewest number of records were made in week 6 when 315 and 199 patients were observed.

report from the Data Monitoring Committee is in preparation.

### Results

During 5 years of accrual, 918 patients with head and neck cancer and 563 with NSCLC were entered by 13 centres (Table I). The peak of entry to the head and neck study was in the second year of the trial but subsequently an average of 12 cases were included each month. Entry to the lung study was at a lower level but maintained at around ten cases per month during the whole period of accrual. A review of the patient characteristics (Table II) shows that apart from a minor trend towards inclusion of patients with more advanced T stage disease in the CHART head and neck group, there was equal randomisation of the known factors which could influence the outcome of treatment of the two tumours.

Two patients entered to the head and neck trial proved ineligible; the stage in one was too early and the other was of unsuitable histology. Both were randomised to the conventional arm. Four patients in the lung trial were also ineligible because one had a performance grade of 2 and 3 showed unsuitable histology. They were randomised equally to the two arms. All six have been included in this interim analysis.

There was a high degree of conformity to protocol, including the delivery of the planned radiotherapy. The size of the treatment fields and the homogeneity of radiation dose through the volumes irradiated were similar in both arms of both trials. Deviations from the protocol were slightly greater in the conventional arms but the differences between the treatment groups were small and to be expected when a course of radiotherapy given over a short period of 12 days is compared with one extending over 42 or 45 days.

The quality assurance team found a high level of precision in treatment delivery in all the centres taking part and in repeat visits the small variance in the values obtained was reduced even further. Detailed results will be reported elsewhere. No errors of any importance were encountered in the comparison of the data with the case notes.

### *Early reactions to radiotherapy*

The reactions within the mucosae of the oral cavity and pharynx appeared sooner and were more severe in the CHART-treated patients. They lasted, however, for a shorter period and subsided as completely as did those in the conventionally treated group (Figure 1a). In contrast, the reactions in the skin, although they appeared sooner, were less marked in the CHART-treated cases and quickly settled (Figure 1b). Intermediate morbidity was assessed at 8 weeks and 3 months, by which time early reactions had largely subsided in both treatment groups.

In the lung study, moderate or severe dysphagia affected 49% of the CHART cases compared with only 19% of those conventionally treated. However, dysphagia settled quickly in the CHART-treated patients and in both arms there was little persistence with this early reaction beyond 6 weeks (Figure 1c).

During the first year, there were five cases of transient myelitis (l'Hermittes syndrome) in the head and neck study; three were in the CHART arm and two in conventionally treated cases. There were six cases in the lung study but all had been treated by CHART.

### *Late reactions to radiotherapy*

In later follow-up, episodes of chondritis, cartilage necrosis or osteonecrosis were reported in 3% of the cases in both arms of the head and neck trial. In the majority, spontaneous healing took place. Analyses of late radiation change observed in the normal tissues in the head and neck region have so far shown no difference between the two arms. In the patients treated for NSCLC, the incidence of radiation fibrosis in the lung and of dysphagia considered caused by radiotherapy has been low and similar in both arms of the study. No case of established radiation myelitis has presented in either study.

### *Tumour control*

In the head and neck trial, disease-free interval was slightly better for CHART with a margin of 3% between the two curves in the life table (Figure 2a). This difference was not statistically significant ($P$=0.33, $\chi^2$=0.95), with a hazard ratio of 0.92 (95% confidence interval 0.76–1.11). There was no evidence that there was a difference in the size of treatment effect in different subgroups defined by primary site, tumour grade, nodal status, age, performance status or sex. However, there was evidence that CHART was more effective than conventional radiotherapy with increasing tumour stage ($\chi^2$ for trend=3.40, $P$=0.065) (Figure 2b–d).

In the lung trial, complete tumour regression was observed in 34% of CHART patients and 29% of the conventionally treated patients. There was a trend for the improved tumour control to be maintained with CHART but this did not reach statistical significance ($P$=0.15, $\chi^2$=2.04).

**Figure 2** Head and neck cancer. Life tables showing the disease-free interval in patients treated by CHART (——) and by conventional radiotherapy (- - -). (**a**) All cases. (**b**) T1 and T2. (**c**) T3. (**d**) T4.

**Table III** Principal cause of death

| *Principal cause of death* | *Head and neck* *CHART* | *Head and neck* *Conventional* | *Non-small-cell lung cancer* *CHART* | *Non-small-cell lung cancer* *Conventional* |
|---|---|---|---|---|
| Primary tumour | 81 (36%) | 62 (45%) | 136 (62%) | 96 (60%) |
| Lymph node metastases | 17 (8%) | 5 (4%) | 49 (22%) | 37 (23%) |
| Post-radiation damage | 1 | 0 | 3 (1%) | 3 (2%) |
| Distant metastases | 39 (17%) | 19 (14%) | 29 (13%) | 21 (13%) |
| Coincidental disease | 72 (32%) | 43 (31%) | 0 | 0 |
| Complications of other treatment | 11 (5%) | 7 (5%) | 3 (1%) | 2 (1%) |
| Unknown | 2 (1%) | 3 (2%) | 0 | 0 |
| Total | 223 | 139 | 220 | 159 |

*Survival*

Among the head and neck patients, 362 deaths have occurred so far, whereas 379 of those with lung carcinoma have died (Table III). Death due to the primary tumour was the main cause of death in both trials but 32% of the deaths in the head and neck trial were due to coincidental disease and 5% to complications of other treatment given subsequently. In the lung study, only 13% of deaths were accounted to coincidental disease and 1% due to complications of other treatment.

In the head and neck study, the overall survival showed no difference between the two groups, but in the NSCLC trial there was improved survival for the CHART-treated patients (Figure 3a) with a hazard ratio of 0.75 (confidence interval 0.61–0.93, $P=0.006$, $\chi^2=7.63$). At 2 years, 30% of the CHART-treated patients were alive and 20% of those treated conventionally. There was a trend towards CHART being more effective with more advanced tumour stage but this did not reach statistical significance ($P=0.29$, $\chi^2=1.13$) (Figure 3b–d).

### Discussion

When the CHART regimen was introduced, it was suggested that early reactions in both the skin and the mucosa would be more severe than with conventional treatment. Previously reported clinical trials of accelerated radiotherapy which were completed in 2 weeks using a conventional 2 Gy dose fraction in head and neck cancer resulted in mucosal reactions of great severity which in some cases did not heal and led on to necrosis (Peracchia and Salti, 1981; Olmi *et al.*, 1990). The use of a small dose per fraction (1.5 Gy) appeared to ameliorate the acute mucosal reactions which, although they appeared earlier and were more troublesome than with conventional treatment, settled sooner, with no more cases of persistence than with conventional treatment.

Of special interest was the diminished reaction in skin. Completion of all radiotherapy before significant reaction was visible in the skin may have increased tolerance because when compensatory proliferation in the normal skin occurred it could proceed without inhibition by further irradiation. A similar finding has now been reported in the skin of pigs (Hopewell and Van Den Aardweg, 1991). The use of CHART in those patients where the full thickness of skin must be irradiated has given a well-tolerated reaction unlike that normally observed with conventional radiotherapy (Dische, 1992).

Transient myelitis after radiotherapy is not associated with any long-term problem (Jones, 1964). It appeared to be associated with CHART in the lung trial but the distribution in the head and neck trial was exactly according to randomisation.

In both trials, the late changes owing to radiation so far observed have been similar in both arms. Further follow-up will show whether the diminished late change associated with CHART and demonstrated in the pilot study will be observed in the randomised trials (Saunders *et al.*, 1991).

For this interim report the disease-free survival of the head and neck patients is reported. As distant metastasis was usually associated with failure in the primary tumour and/or nodes, this closely parallels disease-free probability within the irradiated volume. The definitive report to be produced when the data mature will fully detail the outcome in these patients.

In the conventionally treated arms of the trials, the predicted tumour-free survival of 45% for the head and

**Figure 3** Non-small-cell lung cancer. Life tables showing the overall survival of patients treated by CHART (——) and by conventional radiotherapy (- - -). (a) All cases. (b) T1 and T2. (c) T3. (d) T4.

neck cases was exactly reproduced in the randomised trial while in patients with lung carcinoma there was 20% survival at 2 years compared with a predicted 15%. Disease-free interval was considered the most sensitive indicator of the treatment effect in the head and neck cases; it was almost identical to recurrence rate within the treatment volume; distant metastasis without local failure was rare. In NSCLC, the measurement of local response and detection of recurrence is more difficult to achieve and thus survival was considered to be the principal end point. Where there has been intensive study of primary tumour control in NSCLC, the complete clearance of tumour has been a strong predictor of survival (Saunders, 1991).

Patients presenting with head and neck cancer and those with lung cancer commonly have a history of heavy cigarette smoking and often of high alcohol consumption. Their survival is therefore prejudiced not only by the presence of the tumour for which they are being treated but by the other conditions associated with their lifestyle. The difference between the trials in the incidence of death due to coincidental disease can, in part, be related to the longer survival of the head and neck patients. The greater possibility of a salvage procedure for recurrence of metastases – surgery and cytotoxic chemotherapy – accounts for the 5% incidence of death due to complications of other treatment of which there were only 1% in the patients with lung cancer. Deaths considered due to radiotherapy were rare in both studies.

The trend for improved local tumour control by CHART compared with conventional treatment with increasing stage of disease occurred also in the pilot study of CHART in head and neck cancer (Saunders *et al*., 1991). The data on which this interim report is based were obtained on the same day as case accrual ceased. With further follow-up considerably more data will be available for subgroup analysis and to test the view that patients with advanced stage disease obtained greater benefit with CHART than those with tumours at earlier stages.

Tumour control in the earlier stages of head and neck cancer appeared in both arms of the trial. The choice of CHART for such patients may still be appropriate if a reduction in late morbidity is demonstrated and if the observation that surgery after CHART is easier than after conventional radiotherapy is confirmed in further follow-up. It was apparent that, although patients were prepared to be randomised, a large majority found the completion of all treatments in a 12 day period preferable to daily attendance over a period of 6 to 7 weeks even though a stay in a hostel or ward might be required for the three times a day treatment. (M Leslie, S Dische and MI Saunders, in preparation). The patients' choice may therefore influence the selection of treatment schedule towards CHART.

Sophistication in the delivery of radiotherapy now available using conformal techniques may allow higher doses to be achieved using CHART at the site of gross tumour without increase in morbidity. Further, it may be possible to combine the accelerated treatment with cytotoxic chemotherapy. Using these approaches, additional margins of benefit may be achieved. The CHART regimen may also be applied with advantage to tumours at other sites.

To achieve completion of CHART in 12 days, the total dose was reduced to 54 Gy. It was originally anticipated that the benefit of overcoming tumour cell repopulation would more than compensate for a reduction in the total radiation dose of 12 Gy to the head and neck tumours and 6 Gy to the lung carcinomas. The results of the randomised controlled clinical trials confirm this view and show that the disadvantage of a small reduction in total dose can be outweighed by the benefit of reduced overall time. The differences in result seen between the two trials may be related to the 18% reduction in total dose required to give CHART to the head and neck cases and the 9% for those with lung cancer.

The potential for tumour cells to proliferate rapidly may occur at other sites and have a wide importance in oncology. After surgical resection of tumour, any residual cells may proliferate rapidly in the well-vascularised post-surgical bed. Between cycles of cytotoxic chemotherapy residual tumour cells may be expected to divide rapidly. The shortening of overall time associated with dose intensification in cytotoxic chemotherapy should, in itself, give advantage. The evidence gained in patients treated by radiotherapy showing the ability of tumour cells to proliferate rapidly suggests that when the plan of management is for a combination of treatment modalities – surgery, radiotherapy and cytotoxic chemotherapy – rest intervals between each should be kept as short as possible. Once cancer treatment commences, the greatest success may follow the completion of all its phases in the shortest overall period of time.

### References

BEGG AC, MCNALLY NJ, SHRIEVE DC AND KARCHER HA. (1985). A method to measure the duration of DNA synthesis and the potential doubling time from a single sample. *Cytometry*, **6**, 620–626.

BENNETT MH, WILSON GD, DISCHE S, SAUNDERS MI, MARTINDALE CA AND O'HALLORAN A. (1992). Tumour proliferation assessed by combined histological and flow cytometric analysis: implications for therapy in squamous cell carcinoma in the head and neck. *Br. J. Cancer*, **65**, 870–878.

CHARBIT A, MALAISE EP AND TUBIANA M. (1971). Relation between the pathological nature and the growth rate of human tumors. *Eur. J. Cancer*, **7**, 307–315.

DENEKAMP J. (1982). *Cell Kinetics and Cancer Therapy*. Thomas: Springfield, IL.

DISCHE S. (1992). The clinical science of radiation oncology. *Radiother. Oncol*., **28**, 93–107.

DISCHE S AND SAUNDERS MI. (1995). Clinical fractionation studies. In *Oxford Textbook of Oncology*, Peckham M, Pinedo HM and Veronesi U. (eds), pp 796–810. Oxford Medical Publications: Oxford.

FOWLER JF. (1989). The linear quadratic formula and progress in fractionated radiotherapy. *Br. J. Radiol*., **62**, 679–694.

HOPEWELL JW AND VAN DEN AARDWEG GJMJ. (1991). Studies of dose-fractionation on early and late responses in pig skin: a reappraisal of the importance of the overall treatment time and its effects on radiosensitization and incomplete repair. *Int. J. Radiat. Oncol. Biol. Phys*., **21**, 1441–1450.

HORIOT JC, LE FUR R, N'GUYEN T, CHENAL C, SCHRAUB S, ALFONSI S, GARDANI G, VAN DEN BOGAERT W, DANCZAK S, BOLLA M, VAN GLABBEKE M AND DE PAUW M. (1992). Hyperfractionation versus conventional fractionation in oropharyngeal carcinoma: final analysis of a randomized trial of the EORTC cooperative group of radiotherapy. *Radiother. Oncol*., **25**, 231–241.

INTERNATIONAL COMMISSION ON RADIATION UNITS AND MEASUREMENTS. (1978). Dose specification of reporting external beam therapy with photons and electrons. ICRU Report No. 29. ICRU: Washington, DC.

JONES A. (1964). Transient radiation myelopathy. *Br. J. Radiol*., **37**, 727–744.

OLMI P, CEKKAU E, CHIAVACCI A AND FALLAI C. (1990). Accelerated fractionation in advanced head and neck cancer: results and analysis of late sequelae. *Radiother. Oncol*., **17**, 199–207.

PARMAR MKB AND MACHIN D. (1995). *Survival Analysis: A Practical Approach*. John Wiley: Chichester.

PERACCHIA G AND SALTI C. (1981). Radiotherapy with thrice-a-day fractionation in a short overall time: clinical experiences. *Int. J. Radiat. Oncol. Biol. Phys*., **7**, 99–104.

SAUNDERS MI. (1991). Is the control of the primary tumour worthwhile in non-oat cell carcinoma of the bronchus? *Clin. Oncol*., **3**, 185–188.

SAUNDERS MI, DISCHE S, GROSCH E, FERMONT DC, ASHFORD RFU, MAHER EJ AND MAKEPEACE A. (1991). Experience with CHART. *Int. J. Radiat. Oncol. Biol. Phys.*, **21,** 871-878.

TUBIANA M. (1988). Repopulation in human tumours - a biological background for fractionation in radiotherapy. *Acta. Oncol.*, **27,** 83-88.

VAN DEN KOGEL AJ. (1989). Editorial. Continuous, hyperfractionated, accelerated radiotherapy (CHART). *Radiother. Oncol.*, **16,** 75-77.

WITHERS HR. (1992). Biological basis of radiation therapy for cancer. *Lancet*, **339,** 156-159.